# Optimal Timing of Delivery among Low-Risk Women with Prior Caesarean Section: A Secondary Analysis of the WHO Multicountry Survey on Maternal and Newborn Health

**DOI:** 10.1371/journal.pone.0149091

**Published:** 2016-02-11

**Authors:** Togoobaatar Ganchimeg, Chie Nagata, Joshua P. Vogel, Naho Morisaki, Cynthia Pileggi-Castro, Eduardo Ortiz-Panozo, Kapila Jayaratne, Suneeta Mittal, Erika Ota, João Paulo Souza, Rintaro Mori, WHO Multicountry Survey on Maternal and Newborn Health Research Network

**Affiliations:** 1 Faculty of Medicine, University of Tsukuba, Ibaraki, Japan; 2 Department of Health Policy, National Center for Child Health and Development, Tokyo, Japan; 3 Department of Education for Clinical Research, National Center for Child Health and Development, Tokyo, Japan; 4 UNDP/UNFPA/UNICEF/WHO/World Bank Special Programme of Research, Development and Research Training in Human Reproduction (HRP), Department of Reproductive Health and Research, World Health Organization, Geneva, Switzerland; 5 Department of Social Medicine, National Center for Child Health and Development, Tokyo, Japan; 6 Department of Pediatrics, Ribeirão Preto Medical School, University of São Paulo, São Paulo, Brazil; 7 Center for Population Health Research, National Institute of Public Health, Cuernavaca, Mexico; 8 Family Health Bureau, Ministry of Health, Colombo, Sri Lanka; 9 Department of Obstetrics & Gynecology, Fortis Memorial Research Institute, Gurgaon, India; 10 Department of Social Medicine, Ribeirão Preto Medical School, University of São Paulo, São Paulo, Brazil; University of Helsinki, FINLAND

## Abstract

**Objective:**

To investigate optimal timing of elective repeat caesarean section among low-risk pregnant women with prior caesarean section in a multicountry sample from largely low- and middle-income countries.

**Design:**

Secondary analysis of a cross-sectional study.

**Setting:**

Twenty-nine countries from the World Health Organization Multicountry Survey on Maternal and Newborn Health.

**Population:**

29,647 women with prior caesarean section and no pregnancy complications in their current pregnancy who delivered a term singleton (live birth and stillbirth) at gestational age 37–41 weeks by pre-labour caesarean section, intra-partum caesarean section, or vaginal birth following spontaneous onset of labour.

**Methods:**

We compared the rate of short-term adverse maternal and newborn outcomes following pre-labour caesarean section at a given gestational age, to those following ongoing pregnancies beyond that gestational age.

**Main Outcome Measures:**

Severe maternal outcomes, neonatal morbidity, and intra-hospital early neonatal mortality.

**Results:**

Odds of neonatal morbidity and intra-hospital early neonatal mortality were 0.48 (95% confidence interval [CI] 0.39–0.60) and 0.31 (95% CI 0.16–0.58) times lower for ongoing pregnancies compared to pre-labour caesarean section at 37 weeks. We did not find any significant change in the risk of severe maternal outcomes between pre-labour caesarean section at a given gestational age and ongoing pregnancies beyond that gestational age.

**Conclusions:**

Elective repeat caesarean section at 37 weeks had higher risk of neonatal morbidity and mortality compared to ongoing pregnancy, however risks at later gestational ages did not differ between groups.

## Introduction

Over the last two decades, rates of caesarean section (CS) have increased around the world [1]. Approximately 15% of births worldwide are by CS [2], and CS rates exceed 30% in several high-income countries and Latin American countries [1]. This trend has led to an increase in the number of post-CS pregnancies. Pregnant women with prior CS and their fetuses are at higher risk of maternal/perinatal complications, including placenta accreta, uterine rupture, blood transfusion, hysterectomy, and maternal/fetal death [3–6]. Even in many sub-Saharan African countries where CS rates remain relatively low, proportionately more women are gaining access to CS [7], and the safety of post-CS pregnancies is a growing concern [8].

The safest mode of childbirth for pregnant women with prior CS remains a matter of debate. The birth plan options for women with prior CS include elective repeat caesarean section (ERCS) and trial of labour after caesarean delivery (TOLAC). For women who choose to have ERCS, the optimal timing of ERCS is also important. Numerous studies have reported the relationship between increased risk of neonatal complications, especially respiratory disorders, with elective CS before 39 weeks [9–12]. In previous research, optimal timing of ERCS has mainly been discussed in light of reducing neonatal complications, and delaying elective CS to 39 weeks is advocated in several high-income countries [13–16]. However, around 10% of women are reported to go into labour before 39 weeks [17].

Pregnant women with prior CS could be considered as a specific patient group in need of an appropriate birth plan, obstetrical management, and medical resources in order to achieve safe delivery. However, labour and childbirth in women with prior CS in developing countries may present additional challenges, such as differences in CS quality and safety, limited access to medical services, and variable capacity of health facilities. Thus, it is important to explore these factors to increase knowledge on this issue, to develop appropriate birth plans for post-CS pregnancies, and to ensure better care for mothers and their babies.

This study is a secondary analysis of the World Health Organization Multicountry Survey on Maternal and Newborn Health (WHOMCS), a large multicenter cross-sectional survey of deliveries in 29 countries. In this study, we sought to explore the optimal timing of ERCS among low-risk pregnant women with prior CS by assessing the benefits and risks of pre-labour CS at a given gestational age (GA) compared to ongoing pregnancies beyond that GA.

## Materials and Methods

### Study design and data collection

The present study is a secondary data analysis of the WHOMCS. A multistage cluster sampling method was used to select 359 health facilities in two randomly selected provinces and capital cities from 29 countries in Africa, Asia, Latin America and the Middle East. Methodology and implementation of this study have been published in detail elsewhere [18, 19]. In participating facilities, all women who gave birth, had severe maternal morbidity regardless of GA and delivery status, or who died during pregnancy, delivery or within seven days postpartum, were recruited during the study period from 1 May 2010 to 31 December 2011. Data collection took place at the individual and facility levels. Trained medical staff at these facilities retrieved information from medical records, including individual data on demographics and reproductive characteristics, medical conditions during pregnancy, birth outcomes, complications, and received interventions. Data were collected without any personal identifiers of the study participants, and no information was obtained directly from the study participants. Hence, individual informed consent from the study participants was considered not necessary and was not obtained. Characteristics of each health facility capacity, such as the capabilities of the infrastructure, essential and comprehensive obstetric and neonatal healthcare services, and the facility’s capability to identify and manage severe complications, were obtained through an institutional survey that was completed by the head of the facility or the obstetrics department. Data were collected for two months in facilities with more than 6,000 deliveries per year, and for three months in facilities with less than 6,000 deliveries per year. All data was anonymized and de-identified prior to analysis. The UNDP/UNFPA/UNICEF/WHO/World Bank Special Programme of Research, Development and Research Training in Human Reproduction (HRP) Specialist Panel on Epidemiological Research reviewed and approved the study protocol for technical content. This study was approved by the WHO Ethical Review Committee and the relevant ethical clearance mechanisms in all countries (protocol ID: A65661; date of approval 27 October 2009).

### Study population

**Fig 1:**
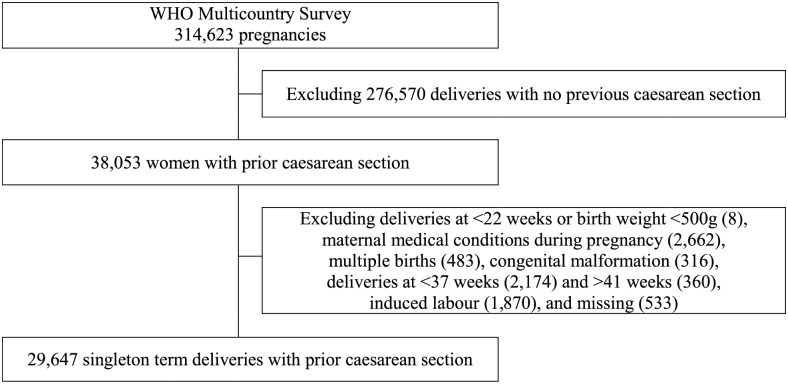
Flow chart of study population.

The population of interest in the present study is women with prior CS who had no pregnancy complications in their current pregnancy and gave birth to a term (i.e. 37 to 41 weeks) singleton (live birth or stillbirth). Of the 314,623 women who participated in the WHOMCS, a total of 38,053 women had a prior CS. We excluded women with medical conditions during pregnancy and childbirth, including severe anaemia (defined as haemoglobin (Hb) <7mg/dl), malaria or dengue fever, preeclampsia/eclampsia, and other conditions, such as disease or injury affecting the heart, lungs, kidney or liver (n = 2,622). In addition, multifetal births (n = 483), congenital malformations (n = 316), deliveries with induction (n = 1,870), and women with missing data on gestational age, onset of labour, fetal presentation, birth weight, and either infant status at birth or seventh day of life (n = 533), were not included in the analysis. As shown in Fig 1, the final analytic sample comprised 29,647 women with prior CS, of whom in the current pregnancy 13,576 had pre-labour CS and 16,071 delivered following spontaneous onset of labour.

### Variables and definitions

Gestational age was reported in WHOMCS by completed weeks. To explore the benefits and risks of repeat CS in the absence of labour at a given GA, it is necessary to compare the rate of adverse outcomes of “pre-labour CS” (i.e. repeat CS in the absence of labour) at a given GA to all “ongoing pregnancies” beyond that GA (i.e. all deliveries at a later GA). We assigned to the pre-labour CS group those women who underwent repeat CS at a given GA in the absence of labour. Those who had spontaneous labour at the same GA were excluded from this group. By excluding these women, we defined the pre-labour CS group as a proxy group of women who had ERCS at the given GA. We also assigned women with ongoing pregnancies who delivered at a later GA to the ongoing pregnancy group, and they would either undergo pre-labour CS or spontaneous labour resulting in intra-partum CS or vaginal birth after caesarean (VBAC) at a later GA. We also defined intra-partum CS as CS after spontaneous onset of labour. In order to obtain the most similar proxy for a group of women who were scheduled for ERCS, we excluded women who had induction of labour from our analysis, since having induction of labour means that those women were scheduled for TOLAC.

In this analysis, we assessed adverse maternal and perinatal outcomes that have previously been described [19–21]. Severe maternal outcomes (SMO) were defined as maternal death or maternal near-miss (MNM) cases that occurred on or before the eighth day postpartum. MNM was defined as women with life-threatening conditions (i.e., failure or dysfunction of any of the vital organ systems including circulatory, respiratory, cardiac, renal, hepatic, central nervous, metabolic and haematological) who almost died but survived pregnancy, childbirth or abortion, and were identified by the WHO criteria (S1 Table) [22].

Adverse neonatal outcomes included early neonatal morbidity and mortality. Neonatal morbidity or neonatal near-miss cases were identified based on management criteria proposed elsewhere [23], which measure the need or use of any of the following lifesaving interventions until the seventh day of neonatal life: any intubation, nasal continuous positive airway pressure, surfactant administration, cardio-pulmonary resuscitation (cardiac massage), any surgery, use of any vasoactive drug, use of any blood products, anticonvulsants, phototherapy in the first 24 hours after birth, steroids to treat refractory hypoglycemia, or therapeutic intravenous antibiotics [23]. Intra-hospital early neonatal mortality (IHENM) was defined as the death of a liveborn neonate in participating facilities within the first week of life or before hospital discharge.

Covariates included in the analysis were: maternal age (<20, 20–34, ≥35), maternal education (≤6, 7–12, >12 years), marital status (single or married/cohabiting), number of prior CS (1, 2, ≥3), health facility capacity (high, medium, low) and countries’ Human Development Index (HDI) based on the 2012 rankings (very high and high, medium, low). In the WHOMCS, detailed information regarding the participating health facilities’ characteristics and capacities was collected using a structured questionnaire. Using these data, facility capacity index was created and calculated as the total score of available services comprising six categories: standard of building/basic services, medical services, emergency obstetric services, laboratory tests, hospital practices, and human resources. Based on its facility capacity index, each facility was further categorized into low, medium, and high [19].

### Analysis and statistical methods

We described the characteristics of the study population, GA at birth, and mode of delivery. We compared the risks of adverse maternal/neonatal outcomes between the pre-labour CS group and the ongoing pregnancies group at a given GA. For instance, we compared pre-labour CS at 37 weeks with deliveries after 37 weeks, pre-labour CS at 38 weeks with deliveries after 38 weeks, and pre-labour CS at 39 weeks with deliveries after 39 weeks. Neonatal morbidity, IHENM, and SMO in the pre-labour CS group at a given GA were calculated by dividing the number of events at that GA by the number of total deliveries at the same GA. Neonatal morbidity, IHENM, and SMO in the ongoing pregnancy group beyond that GA were calculated by dividing the number of events after that GA by the number of total deliveries after that GA. These measures allowed us to compare the risk of adverse outcomes between different gestational lengths, as well as between delivery and continuation of pregnancies at a given GA [24–27]. Crude odds ratios were adjusted for study design (i.e. health facilities as sampling units and countries as strata). We fitted multilevel logistic regression models with random effects of health facilities and adjusted for maternal age, educational level, marital status, number of previous CS, health facility capacity, and countries’ HDI. We reported all adjusted odds ratios with corresponding 95% confidence intervals (95% CI). Missing values were excluded from all logistic regression models. Statistical analysis was conducted using Stata/MP version 13.0 (StataCorp LP, College Station, Texas, USA).

## Results

Data were analyzed from 29,647 women who had CS in their previous pregnancy, had no pregnancy complications in the index pregnancy, and gave birth to a term singleton (Fig 1).

Table 1 shows the characteristics of all women included in this study. The majority of women were aged between 20–34 years (79.0%), married or cohabiting (93.1%), and had one prior CS (77.8%). Approximately half of the women had spontaneous onset of labour, and 35.9% of all women delivered by intra-partum CS. Most women delivered either at 38 or 39 weeks (33.8% and 27.0%). About 40% of women who delivered were in very high and high HDI countries, and 46.8% of deliveries took place at health facilities with medium capacity.

**Table 1:** Characteristics of study population.

Characteristics	No. of women	%
Maternal age (n = 29,647)		
≤19 years	779	2.6
20–34 years	2,3421	79.0
≥35 years	5,447	18.4
Marital status (n = 29,430)		
Single	2,034	6.9
Married/cohabiting	27,396	93.1
Education (n = 29,647)		
≤6 years	6,101	20.6
7–12 years	15,062	50.8
>12 years	8,484	28.6
Number of previous CS (n = 29,647)		
1	23,064	77.8
2	5,413	18.2
≥3	1,170	4.0
Gestational age at delivery (n = 29,647)		
37 weeks	4,297	14.5
38 weeks	10,028	33.8
39 weeks	8,012	27.0
40 weeks	6,004	20.3
41 weeks	1,306	4.4
Fetal presentation (n = 29,647)		
Cephalic	27,996	94.4
Breech/other	1,651	5.6
Mode of delivery (n = 29,647)		
Pre-labour CS	13,576	45.8
Intra-partum CS	10,656	35.9
Vaginal delivery	5,415	18.3
Facility score (n = 25,812)		
High	9,886	38.3
Medium	12,079	46.8
Low	3,847	14.9
HDI country groups (n = 29,647)		
Very high and high	12,019	40.6
Medium	9,584	32.3
Low	8,044	27.1

CS, caesarean section; HDI, Human Development Index as of 2012

**Fig 2:**
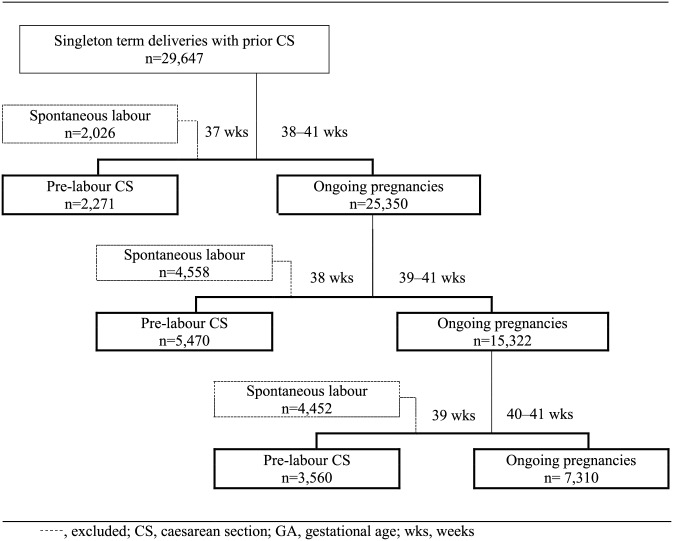
Flow diagram of study group comparing pre-labour CS at a given GA to deliveries at a later GA.

Fig 2 shows the flow diagram of the study groups used for comparison of adverse maternal/neonatal outcomes between pre-labour CS at a given GA and ongoing pregnancies beyond that GA. Table 2 shows adverse maternal and neonatal outcomes for pre-labour CS and ongoing pregnancies at each GA from 37 to 40 weeks. After adjusting for maternal age, education, marital status, number of previous CS, capacity of facility and country HDI, the odds of neonatal morbidity and IHENM were 0.48 (95% CI 0.39–0.60) and 0.31 (95% CI 0.16–0.58) times lower for ongoing pregnancies compared to pre-labour CS at 37 weeks. We also conducted the same analysis by HDI groups; the subgroup analysis also showed higher risk of neonatal morbidity among neonates born by pre-labour CS at 37 weeks consistently across all HDI groups (S2 Table). In addition, a sensitivity analysis was conducted to see whether the results would change in women with only one prior CS and women with more than one prior CS (S3 and S3a Table); however, the results were the same.

**Table 2:** Comparison between pre-labour CS at a given GA and all ongoing pregnancies beyond that GA.

GA	Outcomes	Pre-labour CS	Ongoing pregnancies^§^	Odds ratio^†^ (95% CI)	AOR ^††^(95% CI)
		n (%)	n (%)		
**37 weeks**	**Deliveries**	**2,271**	**25,350**		
	SMO	10 (0.4%)	68 (0.3%)	0.61 (0.30–1.21)	0.70 (0.34–1.42)
	Neonatal morbidity	138 (6.1%)	837 (3.3%)	0.53 (0.41–0.67)**	0.48 (0.39–0.60)**
	IHENM	17 (0.8%)	74 (0.3%)	0.39 (0.22–0.69)*	0.31 (0.16–0.58)**
**38 weeks**	**Deliveries**	**5,470**	**15,322**		
	SMO	16 (0.3%)	37 (0.2%)	0.83 (0.44–1.55)	0.78 (0.38–1.60)
	Neonatal morbidity	192 (3.5%)	473 (3.1%)	0.88 (0.67–1.15)	0.82 (0.66–1.02)
	IHENM	9 (0.2%)	46 (0.3%)	1.84 (0.89–3.77)	2.01 (0.82–4.93)
**39 weeks**	**Deliveries**	**3,560**	**7,310**		
	SMO	5 (0.1%)	21 (0.3%)	2.05 (0.74–5.61)	1.82 (0.61–5.45)
	Neonatal morbidity	110 (3.1%)	226 (3.1%)	1.01 (0.72–1.42)	0.92 (0.68–1.23)
	IHENM	7 (0.2%)	26 (0.4%)	1.83 (0.82–4.07)	1.29 (0.49–3.33)
**40 weeks**	**Deliveries**	**1,857**	**1,306**		
	SMO	5 (0.3%)	5 (0.4%)	1.42 (0.41–4.92)	1.01 (0.21–4.99)
	Neonatal morbidity	56 (3.0%)	51 (4.0%)	1.31 (0.85–2.03)	1.12 (0.70–1.80)
	IHENM	5(0.3%)	5 (0.4%)	1.43 (0.37–5.46)	1.67 (0.38–7.36)

AOR, adjusted odds ratio; CS, caesarean section; GA, gestational age; HDI, Human Development Index as of 2012; IHENM, intra-hospital early neonatal death; SMO, severe maternal outcomes.

^§^ Includes all ongoing pregnancies after given gestational age.

^†^ Calculation of crude odds ratio adjusted for clustering due to survey design.

^††^ Multi-level logistic regression with random effects for health facilities and adjustments made for maternal age, education, marital status, number of previous CS, capacity of facility, and country HDI

*p<0.05

**p<0.01

Description of participating countries’ mode and timing of delivery after prior CS and distribution of adverse maternal/neonatal outcomes were presented in S4 Table and S1 Fig.

## Discussion

To our knowledge, this is the first report to describe timing of delivery among low-risk women with prior CS in a multicountry sample from largely low- and middle-income countries. Our analysis showed wide variation in timing of delivery among women with prior CS at health facilities across the participating countries. We noted that pre-labour CS at 37 weeks had a significantly increased risk of neonatal morbidity and early neonatal mortality in comparison with ongoing pregnancies beyond 37 weeks. We did not find any significant change in the risk of SMO between pre-labour CS at a given GA and ongoing pregnancies beyond that GA.

Optimal timing of ERCS is ideally the GA at which the risk of adverse maternal and neonatal outcomes is minimized. In previous studies, 39 weeks was identified as a threshold GA related to minimized risks of neonatal morbidity and mortality [9–12], and ERCS before 39 weeks was not associated with decreased maternal risk [28]. However, these observational studies assessing risks of ERCS at a certain GA have often compared maternal and neonatal outcomes by GA among women who had ERCS without labour [9–12, 28]. This method discounts risks of adverse events, including those following spontaneous onset of labour which could be avoided by earlier ERCS. Therefore, following several previous studies on optimal timing of delivery and obstetrical interventions [24–27], we compared maternal and neonatal outcomes of pregnancies delivered by pre-labour CS at a certain GA to those receiving expectant management, i.e., pregnancies that were delivered beyond that GA. Using this method, it was indicated that delaying CS beyond 37 weeks significantly reduces morbidity and mortality risk for the neonate.

Delaying ERCS provides time for the fetus to mature, leading to reduced neonatal mortality and morbidity. On the other hand, delaying ERCS increases risk of spontaneous labour before the day of scheduled ERCS, which could lead to life-threatening events such as uterine rupture if emergency CS is not readily available [29]. Earlier ERCS may be beneficial in resource-limited settings to avoid this situation. We therefore expected a higher risk of SMO among women who did not deliver until a later GA. However, this study failed to show that ERCS at an earlier GA reduces risk of SMO. Our results should be interpreted with caution, as we excluded women with pregnancy complications in order to analyse outcomes in low-risk women. Certain complications may reasonably warrant intervention at an earlier GA, and further research for these sub-groups is required. In this analysis, the numbers of the events were small especially at later GAs, and might not be sufficient to test the significance.

Our study had several limitations. First, a lack of information about initial birth plans was a major challenge in determining optimal timing of ERCS, and an inherent limitation of such studies so far. As “scheduling” an ERCS at a certain GA is in itself an intervention, an ideal study design to determine the optimal timing of ERCS would be to compare maternal and perinatal outcomes across the GA for which ERCS was scheduled, regardless of when and how the women actually gave birth. Second, the documented GA was the best obstetric estimate based on local practices. The method of GA assessment for individual women was not captured, and likely varied between health facilities. The error margin for GA may be a factor, as misclassification of GA could affect the results. Third, risk of fetal death due to delayed delivery should also be considered in addition to maternal and neonatal adverse outcomes discussing optimal timing of delivery, since certain types of fetal death could be averted by earlier delivery [30]. However, we could not further evaluate the reduced risk of FD by earlier ERCS as we did not have data on when and how FD occurred. Fourth, due to the nature of the WHOMCS, we could only focus on selected short-term outcomes up to the time of discharge or seventh postpartum day for neonates and eighth postpartum day for mothers. Information on long-term outcomes, such as neonatal death (deaths within the first 28 days of neonatal life) and the ongoing health and development of children, was outside the scope of WHOMCS. Fifth, clinical information regarding the prior CS procedure itself could potentially affect the outcomes of the present pregnancy, however these were not captured in WHOMCS. Sixth, since this is a facility-based study of facilities with CS capacity and over 1,000 deliveries per year, the population in this study may not be representative of populations in countries or other facility types. Lastly, as our analysis explored the association between timing of pre-labour CS and adverse outcomes in a setting where practice is likely to differ between facilities, we cannot deny the possibility that correlation between institutional practice patterns regarding GA at ERCS and their overall performance biased the results. However, since we conducted multilevel analysis which accounted for facility difference, such possible bias was minimized.

## Conclusions

Our analysis showed that ERCS at 37 weeks was associated with a higher risk of early neonatal mortality and morbidity compared to those in later weeks. As there is still uncertainty regarding the relative risks and benefits of scheduling ERCS at a certain GA, these findings should be interpreted with caution. Further studies are warranted to confirm these risks and benefits, and to determine the optimal timing of ERCS for both low- and higher-risk women.

## Supporting Information

S1 Table The WHO maternal near miss criteria. (DOCX)

S2 Table Comparison between pre-labour CS at given GA and all ongoing pregnancies beyond that GA stratified by country groups of Human Development Index. (DOCX)

S3 Table Comparison between pre-labour CS at given GA and all ongoing pregnancies beyond that GA stratified by the number of previous CS. 3a: Comparison of neonatal outcomes between pre-labour CS at 37 weeks gestation and going pregnancies beyond 37 weeks by stratified the number of previous CS. Crude odds ratio and 95% confidence interval.
(DOCX)

S4 Table Number of deliveries with prior caesarean section and birth outcomes by country. (DOC)

S1 Fig Mode of delivery in singleton term low-risk pregnancies with prior CS by country (n = 29,647). (DOC)
