# Supplementary material for: Optimal Timing of Delivery among Low-Risk Women with Prior Caesarean Section: A Secondary Analysis of the WHO Multicountry Survey on Maternal and Newborn Health
Source: PLoS One. 2016 Feb 11;11(2):e0149091. doi: 10.1371/journal.pone.0149091 (PMC4750937; doi:10.1371/journal.pone.0149091)
Supplement: S1 Table — (DOCX) [file pone.0149091.s001.docx]

| S1 Table. The WHO maternal near miss criteria: a woman presenting with any of the following life-threatening conditions and surviving a complication that occurred during pregnancy, childbirth, or within 42 days of termination of pregnancy should be considered as a maternal near miss case. | |
| --- | --- |
| **Clinical criteria** |  |
| Acute cyanosis | Loss of consciousness lasting ≥12 hours^e^ |
| Gasping^a^ | Loss of consciousness AND absence of pulse/heart beat |
| Respiratory rate >40 or <6 /min | Stroke^f^ |
| Shock^b^ | Uncontrollable fit/total paralysis^g^ |
| Oliguria non responsive to fluids or diuretics^c^ | Jaundice in the presence of pre-eclampsia^h^ |
| Clotting failure^d^ |  |
| **Laboratory-based criteria** |  |
| Oxygen saturation <90 % for ≥60 minutes | pH <7.1 |
| PaO2/FiO2 <200 mmHg | Lactate >5 |
| Creatinine ≥300 μmol/l or ≥3.5 mg/dl | Acute thrombocytopenia (<50 000 platelets) |
| Bilirubin>100 μmol/l or > 6.0 mg/dl | Loss of consciousness AND the presence of glucose and ketoacids in urine |
| **Management-based criteria** |  |
| Use of continuous vasoactive drugs^i^ | Intubation and ventilation for ≥60 minutes not related to anaesthesia |
| Hysterectomy following infection or haemorrhage | Dialysis for acute renal failure |
| Transfusion of ≥5 units red cell transfusion | Cardio-pulmonary resuscitation (CPR) |
| ^a^ Gasping is a terminal respiratory pattern and the breath is convulsively and audibly caught. ^b^ Shock is a persistent severe hypotension, defined as a systolic blood pressure <90 mmHg for ≥60 minutes with a pulse rate at least 120 despite aggressive fluid replacement (>2l). ^c^ Oliguria is defined as an urinary output <30 ml/hr for 4 hours or <400 ml/24 hr. ^d^ Clotting failure can be assessed by the bedside clotting test or absence of clotting from the IV site after 7–10 minutes. ^e^ Loss of consciousness is a profound alteration of mental state that involves complete or near-complete lack of responsiveness to external stimuli. It is defined as a Coma Glasgow Scale <10 (moderate or severe coma).  ^f^ Stroke is a neurological deficit of cerebrovascular cause that persists beyond 24 hours or is interrupted by death within 24 hours. ^g^ Condition in which the brain is in a state of continuous seizure. ^h^ Pre-eclampsia is defined as the presence of hypertension associated with proteinuria. Hypertension is defined as a blood pressure of at least 140 mmHg (systolic) or at least 90 mmHg (diastolic) on at least two occasions and at least 4–6 h apart after the 20th week of gestation in women known to be normotensive beforehand. Proteinuria is defined as excretion of 300 mg or more of protein every 24 h. If 24-h urine samples are not available, proteinuria is defined as a protein concentration of 300 mg/l or more (≥1 + on dipstick) in at least two random urine samples taken at least 4–6 h apart. ^i^ For instance, continuous use of any dose of dopamine, epinephrine or norepinephrine. | |
