# Supplementary material for: Optimal Timing of Delivery among Low-Risk Women with Prior Caesarean Section: A Secondary Analysis of the WHO Multicountry Survey on Maternal and Newborn Health
Source: PLoS One. 2016 Feb 11;11(2):e0149091. doi: 10.1371/journal.pone.0149091 (PMC4750937; doi:10.1371/journal.pone.0149091)
Supplement: S2 Table — (DOCX) [file pone.0149091.s002.docx]

| S2 Table. Comparison between pre-labour CS at given GA and all ongoing pregnancies beyond that GA stratified by country groups of Human Development Index. | | | | | | | | | | | | | | | | | | |
| --- | --- | --- | --- | --- | --- | --- | --- | --- | --- | --- | --- | --- | --- | --- | --- | --- | --- | --- |
| GA | Outcomes | Very High and High HDI | | | | |  | Medium HDI | | | | |  | Low HDI | | | | |
|  |  | Pre-labour CS | |  | Ongoing  pregnancies^§^ | |  | Pre-labour CS | |  | Ongoing  pregnancies^§^ | |  | Pre-labour CS | |  | Ongoing  pregnancies^§^ | |
|  |  |  |  |  |  |  |  |  |  |  |  |  |  |  |  |  |  |  |
|  |  | n (%) | |  | n (%) | |  | n (%) | |  | n (%) | |  | n (%) | |  | n (%) | |
| **37 weeks** | **Deliveries** | **1,043** | |  | **10,225** | |  | **710** | |  | **8,190** | |  | **518** | |  | **8,190** | |
|  | SMO | 4 | (0.4%) |  | 25 | (0.2%) |  | 2 | (0.3%) |  | 13 | (0.2%) |  | 4 | (0.8%) |  | 30 | (0.4%) |
|  | Neonatal morbidity *^a,b^* | 58 | (5.6%) |  | 258 | (2.5%) |  | 48 | (6.8%) |  | 280 | (3.4%) |  | 32 | (6.3%) |  | 299 | (4.4%) |
|  | IHENM | 7 | (0.7%) |  | 9 | (0.1%) |  | 5 | (0.7%) |  | 22 | (0.3%) |  | 5 | (1.0%) |  | 43 | (0.6%) |
| **38** | **Deliveries** | **2,559** | |  | **6,062** | |  | **1,845** | |  | **4,844** | |  | **1,066** | |  | **4,416** | |
|  | SMO | 7 | (0.3%) |  | 13 | (0.2%) |  | 3 | (0.2%) |  | 5 | (0.1%) |  | 6 | (0.6%) |  | 19 | (0.4%) |
|  | Neonatal morbidity *^a^* | 82 | (3.2%) |  | 128 | (2.1%) |  | 68 | (3.7%) |  | 142 | (2.9%) |  | 42 | (4.0%) |  | 203 | (4.7%) |
|  | IHENM | 3 | (0.1%) |  | 4 | (0.1%) |  | 3 | (0.2%) |  | 13 | (0.3%) |  | 3 | (0.3%) |  | 29 | (0.7%) |
| **39 weeks** | **Deliveries** | **1,640** | |  | **2,599** | |  | **1,237** | |  | **2,129** | |  | **683** | |  | **2,582** | |
|  | SMO | 2 | (0.1%) |  | 4 | (0.2%) |  | 1 | (0.1%) |  | 3 | (0.1%) |  | 2 | (0.3%) |  | 14 | (0.5%) |
|  | Neonatal morbidity | 42 | (2.6%) |  | 54 | (2.1%) |  | 42 | (3.4%) |  | 54 | (2.6%) |  | 26 | (3.8%) |  | 118 | (4.7%) |
|  | IHENM | 1 | (0.1%) |  | 1 | (0.0%) |  | 3 | (0.2%) |  | 8 | (0.4%) |  | 3 | (0.4%) |  | 17 | (0.7%) |
| **40 weeks** | **Deliveries** | **740** | |  | **492** | |  | **529** | |  | **341** | |  | **588** | |  | **473** | |
|  | SMO | 2 | (0.3%) |  | NR |  |  | 1 | (0.2%) |  | 2 | (0.6%) |  | 2 | (0.3%) |  | 3 | (0.6%) |
|  | Neonatal morbidity *^c^* | 21 | (2.8%) |  | 11 | (2.2%) |  | 18 | (3.4%) |  | 14 | (4.1%) |  | 17 | (2.9%) |  | 26 | (5.5%) |
|  | IHENM | 1 | (0.1%) |  | NR |  |  | 3 | (0.6%) |  | 2 | (0.6%) |  | 1 | (0.2%) |  | 3 | (0.7%) |
| CS, caesarean section; GA, gestational age; HDI, Human Development Index as of 2012; IHENM, intra-hospital early neonatal death; NR, not reported; SMO, severe maternal outcomes.  ^§^ Includes all ongoing pregnancies after given gestational age.  P<0.05 in *^a^* Very High and High HDI, *^b^* Medium and *^c^* Low HDI countries adjusted chi-square test for study design | | | | | | | | | | | | | | | | | | |
