# Supplementary material for: Optimal Timing of Delivery among Low-Risk Women with Prior Caesarean Section: A Secondary Analysis of the WHO Multicountry Survey on Maternal and Newborn Health
Source: PLoS One. 2016 Feb 11;11(2):e0149091. doi: 10.1371/journal.pone.0149091 (PMC4750937; doi:10.1371/journal.pone.0149091)
Supplement: S3 Table — 3a: Comparison of neonatal outcomes between pre-labour CS at 37 weeks gestation and going pregnancies beyond 37 weeks by stratified the number of previous CS. Crude odds ratio and 95% confidence interval. (DOCX) [file pone.0149091.s003.docx]

| S3 Table. Comparison between pre-labour CS at given GA and all ongoing pregnancies beyond that GA stratified by the number of previous CS. | | | | | | | | | | | | |
| --- | --- | --- | --- | --- | --- | --- | --- | --- | --- | --- | --- | --- |
| GA | Outcomes | Women with one previous CS | | | | |  | Women with ≥2 previous CS | | | | |
|  |  | Pre-labour CS | |  | Ongoing pregnancies^§^ | |  | Pre-labour CS | |  | Ongoing pregnancies^§^ | |
|  |  | n (%) | |  | n (%) | |  | n (%) | |  | n (%) | |
| **37 weeks** | **Deliveries** | **1,428** | |  | **20,067** | |  | **843** | |  | **5,283** | |
|  | SMO | 2 | (0.1%) |  | 42 | (0.4%) |  | 8 | (1.0%) |  | 26 | (0.5%) |
|  | Neonatal morbidity *^a,b^* | 96 | (6.8%) |  | 652 | (3.3%) |  | 42 | (5.0%) |  | 185 | (3.5%) |
|  | IHENM *^a^* | 11 | (0.8%) |  | 54 | (0.3%) |  | 6 | (0.7%) |  | 20 | (0.4%) |
| **38 weeks** | **Deliveries** | **3,759** | |  | **12,675** | |  | **1,711** | |  | **2,647** | |
|  | SMO | 10 | (0.3%) |  | 22 | (0.2%) |  | 6 | (0.4%) |  | 15 | (0.6%) |
|  | Neonatal morbidity | 124 | (3.3%) |  | 394 | (3.1%) |  | 68 | (4.0%) |  | 79 | (3.0%) |
|  | IHENM | 3 | (0.1%) |  | 39 | (0.3%) |  | 6 | (0.4%) |  | 7 | (0.3%) |
| **39**  **weeks** | **Deliveries** | **2,648** | |  | **6,240** | |  | **912** | |  | **1,070** | |
|  | SMO | 2 | (0.1%) |  | 12 | (0.2%) |  | 3 | (0.3%) |  | 9 | (0.8%) |
|  | Neonatal morbidity | 85 | (3.2%) |  | 189 | (3.1%) |  | 25 | (2.8%) |  | 37 | (3.5%) |
|  | IHENM | 6 | (0.2%) |  | 23 | (0.4%) |  | 1 | (0.1%) |  | 3 | (0.3%) |
| **40 weeks** | **Deliveries** | **1,470** | |  | **1,132** | |  | **387** | |  | **174** | |
|  | SMO | 3 | (0.2%) |  | 4 | (0.4%) |  | 2 | (0.5%) |  | 1 | (0.6%) |
|  | Neonatal morbidity | 45 | (3.1%) |  | 46 | (4.1%) |  | 11 | (2.9%) |  | 5 | (2.9%) |
|  | IHENM | 4 | (0.3%) |  | 5 | (0.5%) |  | 1 | (0.3%) |  | NR |  |
| CS, caesarean section; GA, gestational age; IHENM, intra-hospital early neonatal death; NR, not reported; SMO, severe maternal outcomes.  ^§^ Includes all ongoing pregnancies after given gestational age.  P<0.05 in *^a^* Women with one previous CS and *^b^* Women with ≥2 previous CS chi-square test adjusted for study design | | | | | | | | | | | | |

| S3a Table. Comparison of neonatal outcomes between pre-labour CS at 37 weeks gestation and going pregnancies beyond 37 weeks by stratified the number of previous CS. Crude odds ratio and 95% confidence interval. | | | | | |
| --- | --- | --- | --- | --- | --- |
|  | Neonatal morbidity | |  | IHENM | |
|  | OR | (95% CI) |  | OR | (95% CI) |
| 1 one prior CS | 0.47 | (0.35-0.62)*** |  | 0.35 | (0.17-0.72)** |
| >1 prior CS | 0.69 | (0.49-0.97)* |  | 0.53 | (0.20-1.38) |
| All women with prior CS | 0.53 | (0.41-0.67)** |  | 0.39 | (0.22-0.69)* |
| CS, caesarean section; CI, confidence interval; IHENM, intra-hospital early neonatal death; OR, odds ratio.  Calculation of Odds ratios were accounted for study deign. *p<0.05 **p<0.01 ***p<0.01 | | | | | |
