# Supplementary material for: Optimal Timing of Delivery among Low-Risk Women with Prior Caesarean Section: A Secondary Analysis of the WHO Multicountry Survey on Maternal and Newborn Health
Source: PLoS One. 2016 Feb 11;11(2):e0149091. doi: 10.1371/journal.pone.0149091 (PMC4750937; doi:10.1371/journal.pone.0149091)
Supplement: S4 Table — (DOC) [file pone.0149091.s004.doc]

| S4 Table. Number of deliveries with prior caesarean section and birth outcomes by country | | | | | | | | | | | | | |
| --- | --- | --- | --- | --- | --- | --- | --- | --- | --- | --- | --- | --- | --- |
| HDI group | Country | Total deliveries | All deliveries with prior CS (%) | | Singleton term deliveries with no pregnancy complications among women with prior CS (n=29,647) | | | | | | | | |
|  |  |  |  |  | n (%) | | 1 prior CS (%) | GA mean (±SD) | Pre-labour CS (%) | Stillbirth (%) | Neonatal morbidity | IHENM | SMO |
| Very high | Japan | 3,537 | 277 | 7.8 | 238 | 6.7 | 85.7 | 37.8 (± 0.77) | 87.4 | NR | 2.5 | NR | 0.4 |
|  | Qatar | 3,950 | 762 | 19.3 | 608 | 15.4 | 69.6 | 38.7 (± 1.03) | 51.3 | 0.2 | 1.2 | 0.2 | 0.3 |
|  | Argentina | 9,807 | 1,860 | 19.0 | 1,523 | 15.5 | 73.3 | 38.7 (± 1.05) | 57.3 | 0.2 | 1.9 | 0.2 | 0.1 |
| High | Mexico | 13,309 | 3,328 | 25.0 | 2,383 | 17.9 | 76.1 | 38.7 (± 1.09) | 34.8 | 0.4 | 4.3 | 0.1 | 0.5 |
|  | Lebanon | 4,044 | 832 | 20.6 | 624 | 15.4 | 62.2 | 38.2 (± 0.82) | 76.1 | 0.2 | 4.3 | 0.2 | 0.5 |
|  | Peru | 15,285 | 2,784 | 18.2 | 2,324 | 15.2 | 77.8 | 38.9 (± 1.07) | 46.0 | 0.3 | 2.1 | 0.0 | 0.1 |
|  | Brazil | 7,058 | 1,352 | 19.2 | 884 | 12.5 | 76.6 | 38.7 (± 1.07) | 68.9 | 0.2 | 1.7 | NR | NR |
|  | Ecuador | 10,245 | 2,469 | 24.1 | 1,659 | 16.2 | 64.5 | 38.8 (± 1.05) | 21.4 | 0.3 | 1.7 | 0.2 | 0.1 |
|  | Sri Lanka | 18,129 | 2,617 | 14.4 | 2,014 | 11.1 | 80.6 | 38.1 (± 0.89) | 80.9 | 0.2 | 5.2 | 0.2 | 0.5 |
| Medium | Jordan | 1,167 | 258 | 22.1 | 193 | 16.5 | 59.6 | 38.5 (± 1.34) | 74.6 | NR | 7.8 | 0.5 | 0.5 |
|  | China | 13,277 | 1,266 | 9.5 | 1,118 | 8.4 | 97.4 | 38.6 (± 0.91) | 69.1 | 0.1 | 1.4 | 0.2 | 0.2 |
|  | Thailand | 8,973 | 1,037 | 11.6 | 871 | 9.7 | 88.4 | 38.1 (± 0.79) | 66.0 | NR | 3.6 | NR | 0.2 |
|  | Mongolia | 7,365 | 718 | 9.7 | 568 | 7.7 | 79.8 | 38.6 (±0.84) | 80.1 | 0.4 | 6.9 | 0.7 | NR |
|  | Occupied Palestinian Territory | 980 | 178 | 18.2 | 122 | 12.4 | 63.9 | 38.4 (± 1.02) | 41.8 | NR | 16.4 | NR | NR |
|  | Paraguay | 3,610 | 774 | 21.4 | 659 | 18.3 | 69.5 | 38.7 (± 1.12) | 38.4 | 0.2 | 2.6 | 0.2 | 0.2 |
|  | Philippines | 10,783 | 1,160 | 10.8 | 1,000 | 9.3 | 73.2 | 38.5(±0.98) | 73.3 | 0.2 | 6.1 | 0.8 | NR |
|  | Vietnam | 15,437 | 2,230 | 14.4 | 2,100 | 13.6 | 92.6 | 39.3 (± 0.92) | 12.2 | NR | 1.0 | NR | 0.1 |
|  | Nicaragua | 6,571 | 893 | 13.6 | 514 | 7.8 | 83.5 | 38.3 (± 0.97) | 56.0 | 0.2 | 4.5 | NR | NR |
|  | India | 31,318 | 2,768 | 8.8 | 2,042 | 6.5 | 86.0 | 38.3 (± 1.03) | 31.5 | 1.7 | 5.9 | 0.9 | 0.4 |
|  | Cambodia | 4,725 | 178 | 3.8 | 159 | 3.4 | 92.5 | 39.3 (± 0.92) | 19.5 | NR | 2.5 | 1.9 | NR |
| Low | Kenya | 20,354 | 2,323 | 11.4 | 1,837 | 9.0 | 78.4 | 38.9 (± 1.11) | 31.4 | 2.3 | 4.6 | 0.6 | 0.4 |
|  | Pakistan | 13,175 | 2,610 | 19.8 | 1,963 | 14.9 | 66.4 | 38.3 (± 1.05) | 54.3 | 1.0 | 4.0 | 1.0 | 0.2 |
|  | Angola | 10,450 | 594 | 5.7 | 486 | 4.7 | 79.6 | 39.3 (± 1.05) | 19.1 | 7.2 | 6.7 | 0.7 | 0.8 |
|  | Nigeria | 12,841 | 986 | 7.7 | 731 | 5.7 | 74.1 | 38.6 (± 1.13) | 56.2 | 1.8 | 4.0 | 0.6 | 1.4 |
|  | Nepal | 11,290 | 616 | 5.5 | 512 | 4.5 | 92.0 | 39.1 (± 1.15) | 46.9 | 1.0 | 7.7 | 0.6 | NR |
|  | Uganda | 10,923 | 920 | 8.4 | 732 | 6.7 | 70.1 | 38.1 (± 0.84) | 17.9 | 2.2 | 4.3 | 0.7 | 0.3 |
|  | Afghanistan | 26,148 | 717 | 2.7 | 594 | 2.3 | 76.3 | 39.1 (± 1.08) | 58.1 | 1.7 | 1.9 | 0.2 | 0.7 |
|  | DR Congo | 8,756 | 1,087 | 12.4 | 868 | 9.9 | 67.3 | 39.4 (± 1.11) | 12.0 | 2.5 | 7.9 | 1.0 | 0.7 |
|  | Niger | 11,116 | 459 | 4.1 | 321 | 2.9 | 85.0 | 39.3 (± 0.93) | 8.4 | 6.5 | 0.3 | 0.3 | NR |
| Overall | | 314,623 | 38,053 | 12.1 | 29,647 | 9.4 | 77.8 | 38.7 (± 1.09) | 45.8 | 0.9 | 3.7 | 0.4 | 0.3 |
| CS, caesarean section; DR Congo, Democratic Republic of the Congo; GA, gestational age; HDI, Human Development Index as of 2012; IHENM, intra-hospital early neonatal mortality; NR, not reported; SMO, severe maternal outcomes. | | | | | | | | | | | | | |
