# Supplementary material for: Optimal Timing of Delivery among Low-Risk Women with Prior Caesarean Section: A Secondary Analysis of the WHO Multicountry Survey on Maternal and Newborn Health
Source: PLoS One. 2016 Feb 11;11(2):e0149091. doi: 10.1371/journal.pone.0149091 (PMC4750937; doi:10.1371/journal.pone.0149091)
Supplement: S1 Fig — (DOC) [file pone.0149091.s005.doc]

S1 Fig. Mode of delivery in singleton term low-risk pregnancies with prior CS by country (n=29,647)


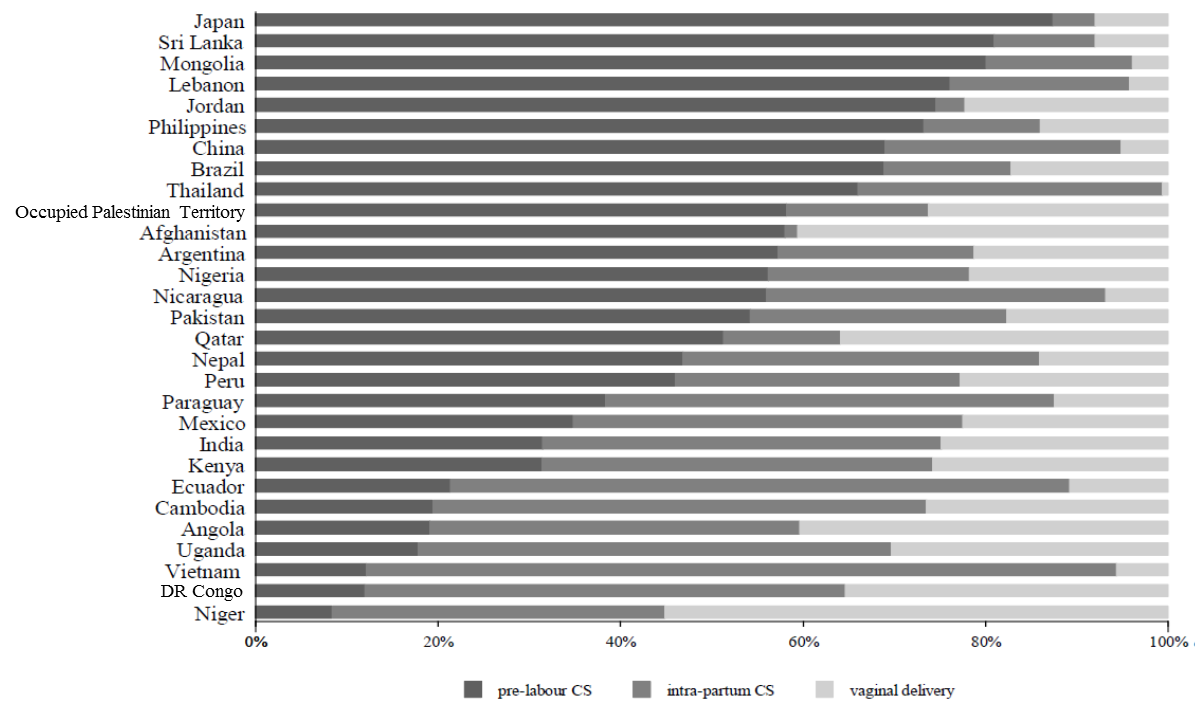


DR Congo, Democratic Republic of the Congo
